# In vitro developmental competence of bovine oocytes: Effect of corpus luteum and follicle size

**Published:** 2015-10

**Authors:** Hamed Karami Shabankareh, Mohammad Hamed Shahsavari, Hadi Hajarian, Gholamali Moghaddam

**Affiliations:** 1 *IVF and ET Laboratory, Department of Animal Sciences, Razi University, Kermanshah, Iran.*; 2 *Department of Animal Sciences, Faculty of Agriculture, University of Tabriz, Tabriz, Iran.*

**Keywords:** Bovine, Development, Oocyte.

## Abstract

**Background:**

Previous studies reported many discrepancies about the effects of corpus luteum (CL) and ovarian follicle size on the developmental competence of oocytes.

**Objective:**

The aim of this study was to investigate the effects of CL and different size of follicle on the developmental potential of bovine oocytes.

**Materials and Methods:**

After ovarian classification based on presence or absence of CL, sample follicles were placed in three groups according to their diameter; small (S; 3–6 mm), medium (M; 6–9 mm), and large (L; 10–20 mm). Collected oocytes in each group were subjected to the in vitro embryo production processes.

**Results:**

Results showed that, the percentages of blastocyst obtained from oocytes originating from small and medium follicles of ovaries bearing a CL (CL+S-oocytes and CL+M-oocytes, respectively) were lower (p<0.001) than those of small and medium follicles of ovaries not bearing a CL (CL-S-oocytes and CL-M-oocytes, respectively) (30.8% and 33.6% vs. 36.9% and 38.7% respectively). Although, the percentages of blastocyst obtained from CL-M-oocytes and CL-L-oocytes were greater (p< 0.001) than those of CL+S-oocytes and CL+M-oocytes. There were no significant differences in the percentages of blastocyst formation between controls (C-oocytes), CL-S-oocytes and CL+L-oocytes.

**Conclusion:**

According to the results of this study, the negative effect of CL on the developmental competence of bovine oocyte depends on the follicle size. Therefore, oocytes originating from large grown follicles were not influenced by negative effects of CL as much as those originating from small and medium follicles did.

## Introduction

There are distinct problems associated with in vitro embryo production (IVEP) of bovine embryos (1). It has been clearly established that oocyte quality (intrinsic quality of primary oocyte coupled to maturation conditions used) determines the rate of blastocyst production (2, 3). These problems include or are derived from the fact that the origin of the oocytes recovered from ovaries obtained from slaughtered animals (stage of estrous cycle, stage of follicular wave, origin of ovaries, etc.) is unknown or heterogeneous and therefore oocyte quality is very variable (4, 5). Previous studies confirmed that there was a relationship between the development of corpus luteum (CL) and the development of follicles which may cause asymmetry in the function of the reproductive organs in dairy cows (6, 7).

The evolution of the CL is associated with viviparity in mammals and is necessary for the production of progesterone [throughout the luteal phase of the estrous cycle to maintain pregnancy (if a conceptus is present) and during pregnancy, to decrease gonadotrophin secretion and prevent behavioral estrous occurring (8, 9). In addition, this small, transient endocrine gland secrets small quantities of oestradiol-17ß, prostaglandins and a number of peptide hormones such as relaxin, oxytocin, oxytocin-related neurophysin-I, vasopressin and inhibin (10). A relationship between the development of CLs and the development of follicles, offers new evidence to support the existence of factors associated with heterogeneity in the developmental competence of oocytes (6). Boediono *et al* and Sugulle *et al* reported some discrepancies about positive and negative effects of CL on oocyte quality too (11, 12).

It appears that there are other factors associated with heterogeneity in the developmental competence of oocytes such as different stages of growth, atresia and different follicle sizes (5, 13, 14). Therefore, another source of heterogeneity in the developmental competence of oocytes is the size of the follicle from which the oocyte is obtained. In the pig as in other species, acquiring meiotic potential and subsequent developmental competence of the oocyte has been shown to be directly correlated to the follicle size (15, 16). In cows, it was reported that the size and the quality of the follicle of origin influence the developmental capacity of bovine oocytes(17, 18). Therefore, interaction between follicle size and the phase of follicular wave affected the efficiency of embryo production (19).

In the present study the effects of CL, different size of follicle and CL by follicle size interactions on the developmental potential of bovine oocytes were investigated.

## Materials and methods

This experimental study was approved and performed under the guidelines of ethics committee for Animal use of Razi University. Unless otherwise stated, all chemicals were purchased from Sigma Chemical Company (St. Louis, MO, USA). This study was performed at Razi University’s IVF & ET Laboratory, located in Kermanshah province; Iran (34^◦^18′ N and 47^◦^3′ E) from January 2013 to March 2013.

**Oocyte collection**

Bovine ovaries were recovered from female adult cows (Holstein friesian) 4-7 years of age with clinically normal reproductive tract after slaughtering. Static ovaries, those associated with pregnant cattle and those that had any pathological lesions such as cystic follicles (>20 mm in diameter) were not included in the study. Collected ovaries were transported to laboratory (within 2 h after slaughter) in a thermos flask in sterile normal salin containing penicillin (100 IU/ml) and streptomycin (50 mg/ml) at 30–35^°^C. To evaluate the developmental potential of oocytes originating from ovaries bearing a CL (CL+-oocytes) or not bearing a CL (CL-oocytes) ovaries were divided into three groups: CL+, CL-, and control ovaries. After ovaries classification based on presence or absence of CL, sample follicles were placed in three groups according to their diameters; small (S; 3–6 mm), medium (M; 6–9 mm) and large (L; 10–20 mm) (20). Cumulus-oocyte complexes (COCs) from these ovarian follicles were aspirated using 18-gauge needles attached to a 10 ml syringe. Therefore, 1330 oocytes were allocated into 7 experimental groups and 6 replicates based on their origin (CL+S-, CL+M-, CL+L-oocytes and CL-S-, CL-M-, CL-L-oocytes and C-oocytes) and their developmental potential were assessed following IVEP. In the control (C-oocytes, without selection based on presence or absence of a CL and follicle size) treatment: oocytes were cultured directly after recovery in the IVP process.

**In vitro maturation (IVM)**

After classification, the COCs were washed three times in the medium in which they were to be cultured. Oocytes were transferred in groups (8-10/group) into 50 ml droplets of IVM culture medium, consisting of Tissue culture medium-199 (TCM 199) supplemented with 0.23 mmol/L sodium pyruvate, 0.02 IU/ml pure human follicle-stimulating hormone (pFSH), 1 μg/ml 17ß estradiol, 50 ng/ml epidermal growth factor (EGF, NO. E-1257, Sigma), 10% (v/v) Fetal calf serum (FCS) and 50 μg/ml gentamicin.

The droplets containing oocytes were covered with pre-warmed (38.5^°^C) mineral oil and incubated for 24 hours (h) at 38.5^º^C in a CO2 incubator (5% CO2 in air, 90–95% relative humidity). After IVM, expansion rate was recorded.

**In vitro fertilization (IVF)**

Frozen bull semen was thawed and prepared by a swim-up procedure (21). After IVM, the oocytes were washed in washing solution and IVF solution twice each. Oocytes were then placed (6-7 COCs/50μl medium) in IVF- Tyrodes albumin lactate pyruvate (TALP) droplets (21). 

The samples of capacitated spermatozoa were added to the oocytes in the droplets for a final concentration of 1×10^6^ spermatozoa/ml. Subsequently, 2 μl of PHE mixture (penicillamine 20 μM, hypotaurine 10, and epinephrine 1 μM) was added to the suspension. Oocytes and spermatozoa were co-incubated for 18 h at 38.5^º^C under 5% CO2 in air with maximum humidity.

**In vitro culture (IVC)**

Approximately 18h post insemination (pi), presumptive zygotes were denuded by gentle vortexing and washed twice in HEPES-TCM 199 containing 4% BSA and once in potassium simplex optimization medium (KSOM) (22).

Groups of 25 to 30 zygotes were cultured in 400 μL of KSOM medium supplemented with 1 mg/mL BSA (KSOM1) overlaid with mineral oil for the first 48 h. Culture droplets were replenished at intervals of 48 h using KSOM medium containing 5% FCS (KSOM2) for the remaining days of culture. Embryonic cleavage was recorded on day 2 (the day of IVF considered as day 0) and blastocyst development was recorded on days 6, 7, 8 and 9 pi (Day 0 - day of IVF) and was expressed as blastocyst per total oocytes and per cleaved embryos.

**Statistical analysis**

Data of this study were collected and the percentage values were arcsine transformed. Analysis of variance was performed by using aov function of Stats package in R software (software statistical package R version 2.15.2). Means were compared using the Tukey HSD test and Student's t-test was used to compare two groups. Differences with a probability value of p ≤ 0.05 were considered to be significant.

## Results

Table I shows the developmental potential of oocytes originating from different follicle size of ovaries bearing a CL (CL+-oocytes) or not bearing a CL (CL--oocytes). In this experiment, follicle size had an effect on percentage of expanded oocytes and S-oocytes were considered to have the lowest percentage of expansion among groups. Presence or absence of CL by follicle size interactions had an effect on the percentage of blastocyst per oocytes and blastocyst per cleaved embryos (p<0.001). The percentage of blastocyst per oocytes of CL+.S-oocytes was the lowest (30.8%) among groups. There was no significant difference (p=0.75) in the percentage of blastocyst between oocytes originating from different follicle size of ovaries not bearing a CL (CL-S, CL-M and CL-L). However, there was heterogeneity in the developmental competence of oocytes originating from different follicle size of ovaries bearing a CL (CL+S, CL+M and CL+L). The percentage of blastocyst obtained from CL-.M-oocytes and CL-.L-oocytes was greater (p< 0.001) than that of CL+.S-oocytes and CL+.M-oocytes. However, there were no significant differences in the percentages of blastocyst formation between C-oocytes, CL-.S-oocytes and CL+L-oocytes. In this experiment, presence or absence of CL by follicle size interactions did not have any effect on expansion and cleavage rates among groups.

**Table I:** Developmental competence (mean±SE) of bovine oocytes originating from different follicle size of ovaries bearing a corpus luteum (CL) or not bearing a CL (replicates = 6)

**Groups**		**Oocytes** **(n)**	**Expansion (%)**	**Cleavage (%)**	**Blastocysts per total oocytes (%)**	**Blastocysts per cleavage (%)**
**CL effect ** **(main effect)** ^ 1^	C CL- CL+	251 531 548	89.2 ± 6.1 89.8± 3.1 85.1 ± 3.2	84.3 ± 3.0 84.2 ± 4.7 78.3 ± 2.8	35.0 ± 0.8 40.2 ± 3.6 31.4 ± 3.4	40.0 ± 0.7 45.7 ± 4.9 38.6 ± 4.2
**Size effect ** **(main effect)** ^ 2^	C S M L	251 435 494 150	89.2 ± 6.1 ^a^ 72.1± 3.6^b^ 81.3 ± 2.4^a^ 90 ± 3.2^a^	84.3 ± 3.0 79.1 ± 2.7 84.4± 2.3 80.5 ± 6.5	35.0 ± 0.8 30.8 ± 3.8 38.3 ± 3.2 41.3 ± 5.4	40.0 ± 0.7 36.2 ± 4.9 41.0 ± 3.9 46.8 ± 7.6
**CL and size effect ** **(Interactions)** ^ 3^	C CL-.S CL-.M CL-.L CL+.S CL+.M CL+.L	251 217 236 78 218 258 72	89.2 ± 6.1 84.8 ± 5.1 88.0 ± 2.1 90.4 ± 4.8 82.4 ± 2.8 83.2 ± 3.5 86.1 ± 6.9	84.3 ± 3.0 81.9 ± 4.9 86.5± 2.6 84.1 ± 12.5 76.1 ± 2.4 82.0± 3.1 76.6 ± 5.5	35.0± 0.8^b^ 36.9 ± 4.0^ab^ 38.7 ± 5.3^a^ 40.4 ± 8.9^a^ 30.8 ± 6.7^c^ 33.6 ± 3.7^bc^ 35.1 ± 6.7^b^	40.0± 0.7^ab^ 39.9 ± 5.4^ab^ 43.6 ± 6.4^a^ 46.1 ± 13.1^a^ 36.8 ± 8.6^b^ 38.8 ± 4.7^b^ 40.7 ± 8.9^ab^

CL: Corpus luteum

Abc; Values in the column with different superscripts differ significantly (p < 0.001). (Student's *t*-test).

1; CL+, CL- and C: Ovaries bearing a CL, not bearing a CL and control ovaries, respectively.

2; S, M, and L: Oocytes originating from small, medium and large follicles, respectively.

3; CL-.S, CL-.M and CL-.L: Oocytes originating from small, medium and large follicles of ovaries not bearing a CL, respectively.

CL+.S, CL+.M and CL+.L: Oocytes originating from small, medium and large follicles of ovaries bearing a CL, respectively.

## Discussion

There are many discrepancies about positive or negative effects of CL on reproductive parameters and the developmental potential of oocytes (23, 24). In addition, another source of heterogeneity in the developmental competence of oocytes is the size of the follicle from which the oocyte is obtained (17). Therefore, the present study was conducted to evaluate the developmental potential of oocytes originating from different follicle size of ovaries bearing a CL (CL+-oocytes) or not bearing a CL (CL-oocytes). The results of the present study showed that presence or absence of CL by follicle size interactions had an effect on the percentage of blastocyst per oocytes and blastocyst per cleaved embryos. 

The percentage of blastocyst formation of oocytes originating from medium and large follicle of ovaries not bearing a CL (CL-M- and CL-L-oocytes) was greater than that of oocytes originating from small and medium follicle of ovaries bearing a CL (CL+S- and CL+M-oocytes) and this pattern was the same in blastocyst per oocytes and blastocyst per cleaved embryos. Furthermore, our results showed that there was a significant difference in the percentage of blastocyst formation between oocytes originating from the same follicle size of ovaries not bearing a CL and ovaries bearing a CL. It was shown that the percentages of blastocyst formation of oocytes originating from small and medium follicle of ovaries bearing a CL (CL+S- and CL+M-oocytes) were lower than that of ovaries not bearing a CL (CL-S- and CL-M-oocytes).

These observations are in agreement with the studies in humans, in which dominant follicles that appeared contralateral to the previous site from where ovulation occurred were healthier than ipsilateral follicles, leading to an enhanced pre-embryo quality in estrous cycles from which oocytes were obtained for IVF (23). Inhibitions of folliculogenesis and ovulation by presence of CL are more frequent on the ovary ipsilateral to the side of the previous ovulation (25, 26). In addition, the results of previous studies in ruminants revealed that the average number of good quality oocytes recovered from ovaries without a CL was comparably higher to the ovaries with a CL, which can be effectively used for IVF (27, 28).

Local paracrine and autocrine influences exerted by the follicles and CL have been reported in many in vitro studies (20, 29, 30). Furthermore, receptors for progesterone and estrogen have been detected in the bovine ovary (31). Indeed, absence of dominance effects during the active phase of the CL, offers new evidence to support the hypothesis of Adams about the existence of suppressive effects of progesterone from the CL on lifespan of dominant follicles in hair breed sheep (32, 6). The mechanism by which progesterone inhibits follicular growth is through suppression of LH pulse frequency, which is critical for continued growth of large follicles (33). It is very well understood that progesterone functions on follicle development by a systemic pathway is through diminishing the frequency of LH pulses. Progesterone may also exert local effects on the growth of large antral follicles, in both luteal and non-luteal ovaries, independent of changes in gonadotrophin secretion (34).

Contreras-Solis *et al* reported the existence of not only a systemic, but also a possible intraovarian effect from the CL on ovine follicular dynamics, with a greater decrease in the number of follicles growing to large sizes in the ovary ipsilateral to the CL (6, 34). The same authors results support the existence of local inhibitory factors released from the CL. Inhibin, which is secreted by the CL of goats and cattle and secreted into ovarian venous blood of ewes, is widely known to affect ovarian follicular growth (6, 35, 36).

As stated earlier, previous studies reported many discrepancies about positive or negative effects of CL on reproductive parameters. For example, in an earlier study in cattle, however, the cleavage rates were found to be higher, and blastocyst production lower in oocytes collected from ovaries without a CL compared to those collected from ovaries with a CL (11). Similarly, in cattle, Boediono *et al* found no difference in the mean number of oocytes per ovary between CL-bearing and non-bearing ovaries and in other studies, the presence or absence of CL did not significantly influence the cleavage rate and blastocyst development (11, 12, 37). 

Data from the present study contrasts with findings in cow that reported higher blastocyst yields have been obtained in vitro from oocytes collected from luteal phase ovaries and from ovaries bearing a CL compared to follicular phase ovaries and those without a CL, probably due to higher progesterone level in the circulation and constant follicular turnover (11, 24, 38). Furthermore, Penitente-Filho *et al *reported that ovaries with CL showed greater numbers of good quality oocytes than ovaries without CL (39).

In the pig as in other species, acquiring meiotic potential and subsequent developmental competence of the oocyte has been shown to be directly correlated to the follicle size (15, 16). In the present study, there was homogeneity in the developmental competence of oocytes originating from different follicle size of ovaries not bearing a CL (CL-S, CL-M and CL-L). However, follicle size had an effect on the developmental competence of oocytes originating from ovaries bearing a CL, where the percentage of blastocyst obtained from CL+L-oocytes was greater than that of CL+S-oocytes. These data contrasts with findings in a study performed in Japan on Black cow that reported the presence of CL within the ovaries does not affect the ovarian follicular dynamics after follicular aspiration and the developmental competence of collected COCs (40). In cows, it was reported that the size and the quality of the follicle of origin influence the developmental capacity of bovine oocytes. In cattle, goats and sheep oocytes originating from large follicles are more competent in terms of in vitro embryo development than oocytes from small follicles (17, 18, 41, 42). Our results confirmed these observations where S-oocytes were considered to have the lowest percentage of expansion among groups. 

Furthermore, in the present study, it appears that all of the CL+-oocytes were not influenced by negative effects of CL and there was heterogeneity in the developmental competence of CL+-oocytes due to the effect of follicle size. It seems that CL+.B-oocytes were not influenced by negative effects of CL as much as CL+.S-oocytes did. A possible reason is that as the follicular diameter increases to approximately 2mm (small follicles) and the oocytes increase in diameter from 110 to 120µm, developmental competency is acquired and the majority of oocytes become capable of supporting fertilization and embryonic development (43). Therefore, it can be hypothesized that CL mostly exerts negative effects on the growth of small growing follicles than large grown follicles.

On the other hand, the concentration of various biochemical constituents of follicular fluid may be changed with advancing the follicular growth and the presence or absence of a CL (44). Shabankareh *et al* suggested that follicular fluid from follicles in the bovine ovaries without a CL is more appropriate to use in oocyte maturation than those from ovaries with a CL because their data showed that the metabolites content of follicular fluid in the ovaries not bearing a CL were higher than those of the ovaries bearing a CL at the time of aspiration (20). These various biochemical constituents of follicular fluid may account for greater percentages of cleavage and blastocyst rates of CL-oocytes than that of CL+-oocytes in the present study. Furthermore, Shabankareh *et al* reported that glucose in the follicular fluid from large follicles being significantly higher compared with that from small and medium follicles in the CL—ovaries (20). Therefore, it appears that presence or absence of CL by follicle size interactions have an effect on oocyte environment and its developmental competence as observed in our study. After formation of the CL, new blood vessels were formed for the development of CL. The CL will receive the greatest rate of blood flow compared with other tissues in the ovary (21). Indeed, the increase in blood flow to the follicular cells in the ovaries without a CL results in an increased supply of gonadotrophins and other systemic biochemicals and hormonal factors necessary for follicular development (45).

In conclusion, the results of this in vitro study clearly demonstrated that CL exerts negative effects on the developmental competence of bovine oocytes. However, oocytes originating from large grown follicles were not influenced by negative effects of CL as much as those originating from small and medium follicles did. In fact, collection of ovaries categorized based on presence or absence of CL and different follicle sizes would be helpful in commercial production of cattle laboratory embryos.

## Note

***This article is extracted from Master thesis (Mohammad Hamed Shahsavari).***
